# Predictors of poor glycemic control and level of glycemic control among diabetic patients in west Ethiopia

**DOI:** 10.1016/j.amsu.2020.04.034

**Published:** 2020-06-01

**Authors:** Mohammed Gebre Dedefo, Selamu Kebamo Abate, Balisa Mosisa Ejeta, Ayana Tadesse Korsa

**Affiliations:** a Clinical Pharmacy Unit, Department of Pharmacy, College of Health Sciences, Wollega University, Nekemte, Oromia, Ethiopia; b Pharmacology Unit, Department of Pharmacy, College of Health Sciences, Wachemo University, Hossana, Ethiopia; c Pharmaceutics Unit, Department of Pharmacy, College of Health Sciences, Wollega University, Nekemte, Oromia, Ethiopia

**Keywords:** Diabetes mellitus, Poor glycemic control, Diabetes knowledge, Ethiopia

## Abstract

**Background:**

Diabetes is a chronic illness that requires continuing medical care and patient self-management education to prevent acute complications and to reduce the risk of long-term complications. Diabetes care is complex and requires that many issues, beyond glycemic control, be addressed. This study aims to assess the level of glycemic control and factors contributing to uncontrolled glycemia among diabetic patients at the Nekemte Referral Hospital, West Ethiopia.

**Methods:**

A cross sectional study was conducted on diabetic patients attending the diabetes clinic of Nekemte Referral Hospital. A total of 252 study participants were included in the study. Data were collected by interviewing patients during hospital visits and reviewing respective databases. The association between dependent and independent variables was assessed using bivariable and stepwise multivariable logistic regression. A variable with a p-value < 0.05 was considered as an independent predictor. A patient's written informed consent was obtained after explaining the purpose and procedures of the study.

**Results:**

Mean age of the participants was 41.7 ± 17.6 years. The majority of the participants (67.1%) had poor knowledge about diabetes. The glycemic rate control was 40.5%; while more than half of the participants (59.5%) had poor glycemic control. On multivariable logistic analysis poor glycemic control was more likely to occur among unemployed (p < 0.001), patients with no family/social support (p = 0.024), duration of diabetes >10 years (p = 0.005), poor knowledge about diabetes (p = 0.012), taking insulin (p = 0.004) and taking metformin plus glibenclamide (p < 0.001).

**Conclusion:**

A finding of this study revealed that a glycemic control of study participants was poor. Thus greater effort is needed to improve glycemic control. Health care professionals should work on improving the adherence to anti-diabetic medications of diabetic patients and knowledge of diabetic patients on diabetes by providing education to the patients during follow up to improve glycemic control.

## Highlights

• The glycemic rate control in the study was 40.5%.
• A finding of this study revealed that a glycemic control of study participants was poor.
• Unemployment, lack of family/social support, duration of diabetes of >10 years, poor knowledge of diabetes, taking insulin and taking metformin plus glibenclamide were identified as factors affecting glycemic control.
• More than three-fourth of the study participants had no access for self-monitoring of blood glucose.
• Two-third of the study participants had poor knowledge about the diabetes.

## 1 Introduction

Diabetes mellitus is a complex, chronic illness requiring continuous medical care with multifactorial risk reduction strategies beyond glycemic control. Ongoing patient self-management education and support are critical to preventing acute complications and reducing the risk of long-term complications. Significant evidence exists that supports a range of interventions to improve diabetes outcomes [[1], [2], [3]].

Diabetes is a major contributor to cardiovascular diseases and is the eleventh common cause of disability worldwide [4]. Undiagnosed or poorly managed diabetes can lead to lower limb amputation, blindness and kidney disease [5,6]. Diabetes also exacerbates major infectious diseases such as TB, HIV/AIDS and malaria [4]. There is also a substantial financial cost to diabetes care as well as costs to the lives of people with diabetes [4,[7], [8], [9]].

Different studies indicated that poor diabetes self-care management behavior, low adherence to medicine, a higher level of anxiety, depression, obesity, literacy status, alcohol and tobacco consumption, treatment strategy, patient's knowledge about disease and treatment, treatment noncompliance, exercise, diet, irregular insulin injection schedules, alcohol use, fear of hypoglycemia, finance, glucose monitoring are associated with poor glycemic control [[10], [11], [12], [13], [14], [15], [16], [17]]. Poor glycemic control also impacts on the health-related quality of life (HRQOL) of patients [[18], [19], [20]].

Studies conducted in Ethiopia reported that poor glycemic control accounts for 60.5–81.9% [[21], [22], [23], [24], [25], [26], [27], [28], [29]]. According to the studies in Ethiopia poor glycemic control was associated with higher body weight [24], knowledge deficit about diabetes [24], poor self-care practice [26], being on insulin therapy [24,27], longer duration of diabetes [27], poor adherence to medication [23,24,26,28] and their educational status [26,29].

Ethiopia's health sector has multiple financing sources, including the government Treasury (federal, regional and woreda/district levels), bilateral and multilateral donors, household out-of-pocket expenditure, international and local nongovernmental organizations (NGOs), private and parastatal employers, and insurance companies. However, more than one third (36%) of the country's health expenditure is bought through out-of-pocket payments made by households [30]. In Ethiopian hospitals, diabetic patients' source of costs was 69.1%, 26.8% and 2.4% for free payment, self-payment, and insurance respectively [31]. This is especially applicable to the funding of insulins (Type 1 and 2 diabetes) as well as an appreciable number of medicines for instance that Type 2 diabetes patients may be prescribed to help control their diabetes and associated complications including oral anti-diabetic agents (one or two including metformin), multiple antihypertensives, statins, low dose aspirin, etc.

This study aimed at assessing the level of glycemic control and factors contributing to uncontrolled glycemia among diabetic patients; hence such type of data will reveal the magnitude of the problem and is important for the care delivery services to fill the gaps to resolve the problem.

## 2 Methods

### 2.1 Study design and population

A cross-sectional study was used. Adult diabetic patients who were on active follow up in DM clinic during the study period at Nekemte Referral Hospital were included in the study. The work has been reported in line with the STROCSS criteria [32]. The Unique Identifying Number (UIN) of this study is researchregistry5338 in Research Registry registration, in accordance with the declaration of Helsinki.

### 2.2 Sample size determination and sampling technique

This work was conducted alongside our previously published paper on self-care practices and we followed the methods of Dedefo et al. [33]. The required sample size was determined by considering the following assumptions for interview questionnaires: Sample size was calculated by taking the proportion of poor glycemic control which was 73.1% on diabetes patients at Diabetes Clinic of Jimma University Specialized Hospital (JUSH) [21] with 95% confidence level and 5% margin of error to get a sample size of 302. The total diabetic patients during our study period were 941. Since the source population consisted of less than 10,000, the sample size was adjusted by using correction formula. The calculated sample size was nf = 229. Considering a 10% non-response rate, 252 diabetic patients were included in the study.

### 2.3 Data collection tool, analysis and interpretation

To collect primary data, questionnaires and interviews were used in this study. The questionnaire was developed after literatures were reviewed thoroughly [[21], [22], [23], [24], [25], [26], [27], [28], [29],[34], [35], [36], [37]]. Data was entered into Statistical Package for the Social Sciences (SPSS) version 20.0 for analysis. Both bivariable and multivariable analyses were done. Odds ratio along with 95% confidence level was estimated to identify factors associated with the outcome variable using multivariable logistic regression analysis. The level of significance was declared at p-value < 0.05 levels.

### 2.4 Definitions of terms

#### 2.4.1 Glycemic control

Glycemic control was assessed by using Fasting Blood Glucose (FBG) level. The glycemic recommendation for non-pregnant adults is between 70 and 130 mg/dl, when the patients FBG was beyond this value we considered as poor glycemic control according to the American Diabetic Association (ADA) [3].

#### 2.4.2 Diabetes knowledge

It is patients' general understanding of diabetes concerning to diet, blood glucose monitoring, foot care, disease complications, sick-day management, proper use of insulin, adverse effects of insulin and factors that influence blood glucose levels. The Diabetes Knowledge Test (DKT) was utilized to assess diabetic patients' general understanding of their disease and treatment recommendations. The DKT was developed and tested for reliability and validity by the University of Michigan scholars and was adapted for the Ethiopian context. DKT consisting of 23 questions has been shown to adequately estimate general patient knowledge of diabetes. The entire questionnaire can be administered to patients who use insulin, but only the first 14 questions apply to patients who do not use this agent. Scores on the DKT were computed for each participant. The score was determined by dividing the number of correct answers by the total number of questions (23 questions for patients taking insulin and 14 for that receiving oral hypoglycemic agents). To assess the level of knowledge of diabetes, we recorded the patients’ level of knowledge into three groups based on their DKT scores as good, acceptable and poor knowledge if their overall score is ≥ 75%, 60–74%, and ≤59% respectively. The scores were used to determine the overall knowledge level [34].

#### 2.4.3 Body mass index (BMI)

BMI was categorized as normal if BMI was <25 kg/m2, overweight if BMI was 25–29.9 kg/m2, and obese if BMI was ≥30 kg/m2 based on the World Health Organization criteria [35].

## 3 Results

This study included 252 participants. More than half of the study participants were male (54.8%). Mean age of the participants was 41.7 ± 17.6 years. Among the participants, 52.0% were in the age range of 30–60 years (Table 1). Out of 252 diabetic patients, 159 (63.1%) were type 1 DM patients while 93 (36.9%) were type 2 DM patients. One hundred fifty-nine (63.1%) patients were taking only insulin and 57 (22.6%) patients were taking only metformin. The majority of the participants (67.1%) had poor knowledge about diabetes. Among the participants, more than half (59.5%) had poor glycemic control (Table 1).

**Table 1:** Socio-demographic and Clinical characteristics of diabetic patients on follow up at Nekemte Referral Hospital, West Ethiopia, from February 20 to May 20, 2016 (n = 252).

Variables	Category	Frequency	Percentage
Sex	Male	138	54.8
	Female	114	45.2
Age	<30	89	35.3
	30–60	131	52.0
	>60	32	12.7
Marital status	Single	74	29.4
	Married	136	54.0
	Divorced	12	4.8
	Widowed	30	11.9
Educational status	No formal education	70	27.8
	Primary school	97	38.5
	Secondary school	48	19.0
	College/University	37	14.7
Occupation	Employed	121	48.0
	Unemployed	131	52.0
Residence	Urban	129	51.2
	Rural	123	48.8
BMI	<18.5 (Underweight)	17	6.7
	18.5–24.9 (Normal weight)	142	56.3
	25–29.9 (Overweight)	50	19.8
	≥30 (Obese)	43	17.1
Family/social support	Yes	64	25.4
	No	188	74.6
Family history of diabetes	Yes	32	12.7
	No	220	87.3
Duration of diabetes	<6	154	61.1
	6–10	69	27.4
	>10	29	11.5
Number of medications taken	<2	138	54.8
	≥2	114	45.2
Access for self-monitoring blood glucose	Yes	26	10.3
	No	226	89.7
Hospitalization due to diabetic-related problem	Yes	53	21.0
	No	199	79.0
Knowledge of diabetes	Good	30	11.9
	Acceptable	53	21.0
	Poor	169	67.1
Anti-diabetic medication	Metformin	57	22.6
	Insulin	159	63.1
	Insulin and Metformin	10	4.0
	Metformin and Glibenclamide	21	8.3
	Glibenclamide	5	2.0
Presence of comorbidities	Yes	75	30.6
	No	175	69.4
Type of diabetes mellitus	Type 1	159	63.1
	Type 2	93	36.9
Glycemic control	≤130	102	40.5
	>130	150	59.5

In this study, variables with p-value < 0.25 like sex, marital status, occupation, family/social support, family history of diabetes, duration of diabetes, access for self-monitoring blood glucose, knowledge of diabetes, anti-diabetic medication and type of DM were entered into multivariable analysis to identify independent predictors of poor glycemic control among diabetic patients (Table 2).

**Table 2:** Bivariable analysis of factors associated with glycemic control among diabetic patients on follow up at Nekemte Referral Hospital, West Ethiopia, from February 20 to May 20, 2016 (n = 252).

Variables	Categories	Glycemic Control		p-value	COR (95% CI) for Poor Glycemic control
		≤130	>130		
Sex	Male	66	72		1.00
	Female	36	78	0.009	1.986(1.184–3.331)
Age	<30	32	57	0.437	1.385(0.609–3.151)
	30–60	56	75	0.918	1.042(0.478–2.271)
	>60	14	18	0.556	1.00
Marital status	Single	26	48	0.046	2.414(1.016–5.737)
	Married	55	81	0.108	1.926(0.866–4.283)
	Divorced	4	8	0.179	2.615(0.644–10.614)
	Widowed	17	13	0.233	1.00
Educational status	No formal education	23	47	0.290	1.557(0.686–3.534)
	Primary school	40	57	0.833	1.086(0.505–2.335)
	Secondary school	23	25	0.668	0.828(0.350–1.962)
	College/University	16	21	0.406	1.00
Occupation	Employed	69	52		1.00
	Unemployed	33	98	0.000	3.941(2.310–6.722)
Residence	Urban	55	74		1.00
	Rural	47	76	0.475	1.202(0.726–1.989)
BMI	<18.5	8	9	0.813	1.00
	18.5–24.9	54	88	0.472	1.449(0.527–3.981)
	25–29.9	21	29	0.716	1.228(0.406–3.708)
	≥30	19	24	0.840	1.123(0.364–3.464)
Family/social support	Yes	34	30		1.00
	No	68	120	0.018	2.000(1.126–3.551)
Family history of diabetes	Yes	16	16		1.00
	No	86	134	0.243	1.558(0.740–3.279)
Duration of diabetes	<6	72	82	0.007	1.00
	6–10	26	43	0.208	1.452(0.812–2.596)
	>10	4	25	0.002	5.488(1.823–16.518)
Number of medications taken	<2	59	79		1.00
	≥2	43	71	0.418	1.233(0.743–2.048)
Access for self-monitoring BG	Yes	6	20		1.00
	No	96	130	0.063	0.406(0.157–1.050)
Hospitalization due to diabetic-related problem	Yes	22	31	0.863	0.947(0.512–1.753)
	No	80	119		1.00
Knowledge of diabetes	Good	21	9	0.002	1.00
	Acceptable	23	30	0.022	3.043(1.176–7.879)
	Poor	58	111	0.001	4.466(1.922–10.375)
Anti-diabetic medication	Metformin	32	25	0.063	1.00
	Insulin	60	99	0.017	2.112(1.143–3.901)
	Insulin and Metformin	2	8	0.050	5.120(0.998–26.274)
	Metformin and Glibenclamide	6	15	0.035	3.200(1.085–9.439)
	Glibenclamide	2	3	0.493	1.920(0.298–12.384)
Presence of comorbidities	Yes	30	47	0.745	1.095(0.633–1.895)
	No	72	103		1.00
Type of diabetes mellitus	Type 1	60	99	0.247	1.359(0.808–2.284)
	Type 2	42	51		1.00

According to the results of multivariable logistic analysis poor glycemic control was more likely to occur among unemployed (AOR = 4.998, 95% CI = 2.579–9.688, p < 0.001), patients with no family/social support (AOR = 2.547, 95% CI = 1.131–5.733, p = 0.024), duration of diabetes >10 years (AOR = 6.359, 95% CI = 1.740–23.242, p = 0.005), poor knowledge about diabetes (AOR = 4.222, 95% CI = 1.378–12.932, p = 0.012), taking insulin (AOR = 3.359, 95% CI = 1.471–7.675, p = 0.004), taking metformin plus glibenclamide (AOR = 12.887, 95% CI = 3.184–52.158, p < 0.001) (Table 3).

**Table 3:** Multivariable analysis of factors associated with glycemic control among diabetic patients on follow up at Nekemte Referral Hospital, West Ethiopia, from February 20 to May 20, 2016 (n = 252).

Variables	Categories	p-value	AOR (95% CI) for Poor Glycemic control
Occupation	Employed		1.00
	Unemployed	0.000	4.998(2.579–9.688)
Family/social support	Yes		1.00
	No	0.024	2.547(1.131–5.733)
Family history of diabetes	Yes		1.00
	No	0.060	2.460(0.962–6.292)
Duration of diabetes	<6	0.015	1.00
	6–10	0.963	0.982(0.465–2.075)
	>10	0.005	6.359(1.740–23.242)
Knowledge of diabetes	Good	0.041	1.00
	Acceptable	0.069	3.210(0.913–11.284)
	Poor	0.012	4.222(1.378–12.932)
Anti-diabetic medication	Metformin	0.002	1.00
	Insulin	0.004	3.359(1.471–7.675)
	Insulin and Metformin	0.107	4.598(0.719–29.394)
	Metformin and Glibenclamide	0.000	12.887(3.184–52.158)
	Glibenclamide	0.713	0.683(0.089–5.230)

## 4 Discussion

This study assessed glycemic control among diabetic patients and factors associated with glycemic control. Our study revealed that more than half of the participants (59.5%) had poor glycemic control. This is comparable with studies done in Northwest Ethiopia (60.5%) [22], Southwest Ethiopia (64%) [23] and Mekelle (61.9%) [25]. However, the glycemic control in this study is better than other studies done in Ethiopia in which poor glycemic control was reported as 73.1% [21], 81.7% [24],81.9% [26], 80% [27], 64.7% [28] and 70.9% [29]. The comparison of our findings with previous studies done in Ethiopia could reflect an improvement in diabetes care services in Ethiopia. However, the glycemic control in this study is poor as compared to studies done in Iran [15] and Korea [36] in which poor glycemic control was reported as 47% and 49.5% respectively. The probable reason for the difference could be as a result of knowledge difference of participants between developing and developed countries, lack of uniform guidelines for assessing glycemic control for physicians to set the cut-off, and health insurance and the difference in health insurance access and coverage at primary care [37]. Inadequate knowledge of patients about diabetes that affects glycemic control in our setting could be related to a lack of health education provision about diabetes to the patients.

This study showed that unemployment was associated significantly with poor glycemic control. This could be because unemployed patients may not be able to afford the cost of the medications and this could affect their adherence which will result in poor glycemic control. This finding is consistent with that reported by other studies from Ethiopia [29], Libya [38] and Korea [39].

The present study revealed that patients with no family/social support were associated significantly with poor glycemic control. Patients with family/social support have improved patients’ adherence to their medication and good adherence will improve glycemic control [[40], [41], [42]], thus patients with no family/social support could have poor glycemic control. Similar results were found in a study conducted in the USA [43].

Patients with longer duration of diabetes had shown a significant association with poor glycemic control. The reason for poor glycemic control in patients with longer duration of diabetes is due to decreased in function of pancreatic over time that will result in subsequent lower levels of secreted insulin and insulin resistance, which is typical in type 2 diabetes, this in turn yields the worsening of glucose control [44]. The present finding is consistent with that reported by other studies from Ethiopia [27], Jordan [16], Tanzania [17], Korea [36,39], Brazil [45], Hawaii [46], Peru [47] and Malaysia [48].

Poor knowledge about diabetes was associated significantly with poor glycemic control. This finding is consistent with other studies done in Ethiopia [24], India [11] and Turkey [49]. This could be explained as patients with poor knowledge about diabetes are less compliant to their medication and self-care practice and this will result in poor glycemic control [16,38,50].

Patients taking insulin alone (AOR = 3.359, 95% CI = 1.471–7.675, p = 0.004) and patients taking metformin plus glibenclamide (AOR = 12.887, 95% CI = 3.184–52.158, p < 0.001) were 3.4 times and 12.9 times more likely to have poor glycemic control as compared to patients taking metformin alone respectively. Similar results were reported from studies done in Ethiopia [21,26,29], Libya [38], Brazil [45] and Jordan [16]. The reason for poor glycemic control in patients taking insulin alone could be because of inappropriate use of insulin as a result of inadequate knowledge of patients on injection sites rotation, lack of knowledge on insulin storage and lack of knowledge on the use of disposable syringe-needles. In the case of patients who were treated by a combination of metformin and glibenclamide means their diabetes condition requires more aggressive treatment because the disease progression and blood glucose will become difficult to control as compared to patients on single metformin in which it will be given during the early course of the disease.

The present study also showed that the proportion of poor glycemic control was higher among type 1 DM patients (62.5%) than type 2 DM patients (54.8%). This is consistent with studies done at the University of Gondar referral hospital [22,28], Saudi Arabia [51] and Venezuela [52]. The reason could be because of the inappropriate use of insulin by the patients for the treatment of type 1 DM.

## 5 Conclusion

In conclusion, a finding of this study revealed that a glycemic control of study participants was poor. Thus greater effort is needed to improve glycemic control. Factors like unemployment, lack of family/social support, duration of diabetes of >10 years, poor knowledge of diabetes, taking insulin alone and taking metformin plus glibenclamide were associated significantly with poor glycemic control. We recommend that health care professionals should work on providing health education to the patients during follow up and providing community service on the knowledge of diabetes, self-care practice and administration of insulin.

## Strength and limitations of this study

This study was the first study conducted at Nekemte referral hospital located in West Ethiopia to determine factors associated with glycemic control among diabetic patients. This study used data from patients’ cards and face to face interviews; this allowed us to have complete information to determine glycemic control and identify associated factors.

The limitations of our study were that we used FBG to evaluate glycemic control because HbA1c determination is not available in the public health sector of Ethiopia. The use of FBG over HbA1c which is more accurate than FBG measurement to evaluate glycemic control may underestimate the prevalence of poor glycemic control. This limitation is not only affecting our study, but also a significant challenge for diabetes control in the country as a whole without access to Hb1Ac measurement. Self-reporting was used for measuring diabetes knowledge. Therefore, the results could be susceptible to bias, which may not reflect the participants’ actual level of diabetes knowledge.

## Ethical approval and consent to participate

Ethical clearance was obtained from the Ethical Review Committee of Wollega University, College of Medical and Health Sciences dated the February 15, 2016; reference number “WU:90,340/ST_1_-49”. This committee wrote a formal letter of permission to Nekemte Referral Hospital to seek its cooperation and access to the patients and data. Permission was obtained from the medical director's office of the hospital. A patient's written informed consent was obtained after explaining the purpose and procedures of the study. The confidentiality of the study participants was secured. Besides, all the responses were kept confidential.

## Availabilitzy of data and materials

Data is available upon request from the corresponding author.

## Author contributions

MGD involved in the conception of the research idea, designing of the study, set the objective, participated in data collection, performed data analysis, interpretation, and drafting of the manuscript; SKA, BME & ATK involved in designing of the study, set the objective, performed data analysis, interpretation, and drafting of the manuscript. All authors approved the manuscript for publication.

## Funding

Not applicable.

## Provenance and peer review

Not commissioned, externally peer reviewed.

## Declaration of competing interest

The authors declare that they have no conflicts of interest.

## Abbreviations

AOR: Adjusted Odds Ratio
BMI: Body mass index
COR: Crude Odds Ratio
DKT: Diabetes Knowledge Test
DM: Diabetes Mellitus
FBG: Fasting Blood Glucose
HbA1c: Glycosylated Hemoglobin
SPSS: Statistical Package for the Social Sciences
USA: United States of America
